# 18:2 Cholesterol Ester is a Novel Prognostic Biomarker of Disease Progression in Multiple Sclerosis

**DOI:** 10.1007/s12035-026-05915-8

**Published:** 2026-06-17

**Authors:** Pascual Torres, Anna Gil Sánchez, Agustín Sancho-Saldaña, Laura Quibus, Eduardo San Pedro Murillo, Emilio Ruiz-Fernández, Silvia Peralta, Maria José Solana, Luis Brieva, Cristina González-Mingot

**Affiliations:** 1 Metabolic Physiopathology Group, University of Lleida, IRBLleida, Av. Alcalde Rovira Roure, 80, 25198 Lleida, Spain; 2 Neuroimmunology Group, Department of Medicine, University of Lleida, IRBLleida, Av. Alcalde Rovira Roure, 80, 25198 Lleida, Spain; 3 Department of Neurology, Hospital Universitari Arnau de Vilanova, 25198 Lleida, Spain

**Keywords:** Multiple sclerosis, Biomarkers, Lipid, Cholesterol, Retrospective study, Disease progression, PIRA

## Abstract

Multiple sclerosis (MS) presents diverse clinical outcomes. Cholesterol esterification (CE) has been implicated as a disease mechanism involved in the remyelination process. We investigated whether cerebrospinal fluid (CSF) 18:2 cholesterol ester (18:2 CE) levels at diagnosis could predict 10-year MS progression. We aimed to determine if CSF 18:2 CE levels correlate with long-term disease progression. In a retrospective case–control single-centre study with 115 MS patients and 57 controls, we conducted targeted lipidomic analysis of CSF. Clinical data, oligoclonal bands, brain and spinal cord MRI, and CSF 18:2 CE levels were collected. Elevated CSF 18:2 CE levels were significantly associated with a higher risk of 10-year MS progression, with an AUC of 0.91 in a multivariable logistic regression model. Patients initially treated with a high-efficacy drug did not show a worse prognosis despite presenting elevated CE18:2 levels at the time of diagnosis. However, patients treated with a high-efficacy drug as a second or third option showed a positive correlation between CE18:2 levels and EDSS at 10 years. CSF 18:2 CE is a potential prognostic biomarker for predicting long-term MS progression, potentially indicating reduced remyelination capacity. These findings hold promise for improved patient management and warrant prospective validation.

**Supplementary Information:**

The online version contains supplementary material available at 10.1007/s12035-026-05915-8.

## Introduction

Multiple sclerosis (MS) is an immune-mediated, chronic inflammatory, demyelinating, and neurodegenerative disease which affects the central nervous system (CNS) and is characterized by multifactorial pathogenesis. It has a very heterogeneous clinical presentation [1] and is the leading cause of disability in young adults [2]. Disease-modifying therapies (DMTs) are highly efficient in reducing the number of relapses in RRMS patients. However, there are no DMTs capable of restoring neurological disabilities or substantially modifying the progression of disability in progressive MS (PMS).

Recent research has confirmed that long-term disability in MS is mostly unrelated to relapses, a phenomenon known as progression independent of relapse activity (PIRA). PIRA had been potentially linked to the neurodegenerative process in RRMS [3]. This silent progression may be widespread in RRMS patients and could be a factor in the clinical symptoms of SPMS when evident clinical worsening occurs. Moreover, some authors proposed that PPMS may simply be a form of SPMS in which the relapsing-remitting phase is clinically silent [4]. Consequently, the pathophysiological events in SPMS may occur in the early phase of the disease and closely correlate with long-term prognosis, with implications for therapeutic decision-making [5].

Around 70% of the cholesterol in the adult brain is linked to myelin, a lipid-rich membrane stack that provides insulation to axons. In the process of brain development, oligodendrocytes are primarily responsible for producing the cholesterol needed for the expansion of the myelin membrane. In the adult brain, all types of cells play a role in maintaining cholesterol balance within the CNS, and this regulation is separate from peripheral sources. Because mammals lack the ability to break down cholesterol, it is either expelled from the brain or recycled locally [6]. CE is formed by the action of Lecithin:cholesterol acyltransferase (LCAT) and is directed to apoE in which it is transported across the CSF [7, 8]. Several authors have suggested that lipid mediators could be involved in autoimmune attacks on neurons [9, 10]. The effective restoration of damaged myelin in individuals with MS is intricately connected to the local metabolism of cholesterol. During demyelinating insults, cholesterol is released from damaged myelin, leading to a reduction in local sterol synthesis due to feedback inhibition [11]. To phagocytose and eliminate myelin debris, microglia/macrophages adopt a pro-inflammatory profile [12].Chronic active MS lesions contain numerous foamy phagocytes with internalized myelin, suggesting suboptimal lipid recycling [13]. Neuropathological studies in MS have emphasized the role of chronically activated microglia in the progression of the disease. Activated microglia contributes to local or compartmentalized inflammation, a phenomenon known as “smouldering”, hindering the remyelination process [4].

Spontaneous remyelination occurs in animal models and likely contributes during the early stages of MS [14]. However, this process requires the shift toward a regenerative environment. During this phase, oligodendrocytes generate myelin membranes as part of the functional repair mechanism [15]. Recent studies show that sterol synthesis in microglia/macrophages was essential for repair after acute demyelination [16]. Failures in the repair process could be due to ongoing toxic neuroinflammation and/or to an inhibitory lesion microenvironment that prevents the differentiation of oligodendrocyte progenitor cells into myelin-forming oligodendrocytes [17]. One strategy for counteracting neurodegeneration is to promote neuroprotection by improving myelin regeneration and thus restoring nerve conduction and metabolic support to the axon [18]. Other studies highlight the importance of detoxifying cholesterol debris into cholesterol esters for the correction of myelin defects as an essential step for remyelination in MS [19]. The esterification of cholesterol is the preliminary step before the formation of lipid droplets, which are the vesicles where cholesterol will be stored, awaiting reuse to restore myelin membranes [13].

While there has been considerable progress in comprehending the pathomechanism of MS, there remains limited knowledge about the underlying cellular and molecular mechanisms influencing smouldering lesion formation and PIRA. Understanding these pathobiochemical markers is crucial, as they can significantly impact therapeutic decisions and provide insight into the relationship between clinical classification and the pathomechanism of MS.

An active area of current research is the search for new biomarkers that will help us to better understand the etiology of MS, aid in its diagnosis and prognosis, and facilitate the implementation of personalized medical treatments [20–22].

Lipidomics is an important resource for the study of multifactorial diseases [23]. Under pathological conditions, cells can undergo metabolic changes that could alter their lipidomes. Such changes can be detected in the cerebrospinal fluid (CSF), as it reflects the brain environment thanks to its close contact with that organ and its nutritional functions [24]. Our group has published an article in which we concluded that the description of the lipidomic profile of the CSF at the time of diagnosis could help us better understand the pathophysiology of MS in its early stages [25]. Subsequent analysis of our unpublished data revealed that 18:2 CE could serve as potential prognostic biomarkers, as they exhibited statistically significant differences in their levels in the cerebrospinal fluid (CSF) of patients with aggressive courses of MS compared to in those with benign courses. However, our research had certain limitations, and these data were obtained from a cohort with a relatively small sample size (*N* = 36). Furthermore, a non-directed approach was followed, which made reproducibility rather limited. Nevertheless, our research also pointed to a pathological mechanism in a certain group of patients. This could also be used to define groups in clinical trials involving drugs aimed at modulating cholesterol esterification.

Our hypothesis is that differences in the levels of cholesterol esters in the CSF of MS patients with more aggressive progressions indicate a deficit in remyelination which aggravates the injury, leading to a higher degree of disability. Absolute quantification by lipidomics, carried out at the time of diagnosis, could be used to predict disease progression in patients.

The main objective of this work was to quantify 18:2 CE in MS patients with varying degrees of progression and to analyze their potential as prognostic biomarkers.

Studying lipids could be an important tool and help to better understand the biological processes that occur at the onset of MS. It could even aid in the diagnosis/prognosis of MS and pave the way for new therapeutic targets such as remyelination.

## Methods

### Study Design and Patients

This was a single-centre retrospective case-control study involving independents groups of 115 MS patients (21 PPMS and 94 RRMS) who were diagnosed using 2010 McDonald’s revised criteria [26] and 57 controls (comprised patients undergoing lumbar puncture due to suspected intracranial hypertension, with inflammatory and demyelinating disorders explicitly excluded). Participants were matched by age and sex. For consistency and contemporary relevance, cases were retrospectively reclassified using McDonald 2017. Exclusion criteria were the presence of any other neurological or systemic autoimmune/inflammatory disease, a neurodegenerative disorder, neoplastic disease, treatment with anticoagulants, or any condition precluding lumbar puncture and/or MRI acquisition. CSF samples were obtained at the time of the diagnosis of the disease, prior to corticosteroid administration, and there was a follow-up period of a minimum of 10 years. The patients included in this study were diagnosed at the multiple sclerosis unit of the Arnau de Vilanova University Hospital in Lleida. Patient follow-up was conducted through biannual visits. Assessment included measurements using the Expanded Disability Status Scale (EDSS) score, neuro-psychological study, and annual brain MRI. We included (1) patients who met the diagnostic criteria for MS based on the 2010 McDonald criteria and (2) patients for whom a cerebrospinal fluid (CSF) sample was available at the time of diagnosis. The EDSS scores of all the patients were validated by a Neurostatus-EDSS-certified physician. Any relapses were recorded by the treating neurologist at the onset of new, or recurrent, MS symptoms lasting for ≥ 24 h.

The primary endpoint was the 10-year disease severity phenotype (Mild vs Severe) defined as:

- Mild: EDSS ≤ 3 with 10 years of evolution without confirmed and sustained disability worsening (CDW) except for random outbreaks and without high-efficacy treatments.
- Severe: EDSS ≥ 6 after 10 years of evolution or with high-efficacy treatments over 10 years.

CDW events were based on EDSS and defined by an increase in EDSS (≥ 1.5 points for patients with a baseline EDSS of zero;  ≥ 1.0 points for patients with a baseline EDSS of 1–5; and by 0.5 points for patients with a baseline EDSS of  ≥ 5.5) which was confirmed by another EDSS assessment made at least 3 or 6 months after the onset of the worsening. We categorized CDW events as either relapse-associated worsening (RAW) or PIRA.

i. RAW was related to a 3- or 6-month CDW event with an onset within 90 days of the investigator-reported relapse (irrespective of the EDSS confirmation)
ii. PIRA was defined as a 3- or 6-month CDW event with either no prior relapse or an onset more than 90 days after the last investigator-reported relapse (irrespective of the EDSS confirmation). In addition, to qualify as a PIRA event, there could be no relapse within 30 days (either before or after) of the EDSS confirmation.

NEDA-3 (no evidence of disease activity 3) was the key secondary endpoint defined as the absence of disease activity across three domains: (i) no relapses; (ii) no MRI activity (no new/enlarging T2 lesions and no gadolinium-enhancing lesions); and (iii) no confirmed disability worsening.

Baseline MRI was acquired on the same 1.5-T scanners using an unchanged, standardized protocol including axial oblique proton density–weighted (PDw), T2-weighted, and T1-weighted sequences acquired before and after gadolinium injection, as well as sagittal/axial/coronal FLAIR and double inversion recovery (DIR) sequences.

The study was evaluated by the local ethics committee of the Arnau de Vilanova University Hospital (Lleida, Spain): CEIC-2719. This was carried out in accordance with the Code of Ethics of the World Medical Association (Helsinki Declaration). Informed consent was obtained from all participants at the time of their lumbar punctures and included a subsection in which we were given the samples for future research. All the patients signed to give their consent.

### Targeted Lipidomics

18:2 CE standards were purchased from Sigma (cat. No. C0289). A Triple Quad 6420 LC/MS Agilent Technologies (TQD) mass detection system was used for sample analysis. The processing of the samples was carried out by the Lipidomics Service of IRBLleida. Lipid extraction was performed in a single-phase method described previously [27] mixing 10 µL of sample with 100 µL of butanol: methanol (1:1) containing internal standards. Samples were vortexed thoroughly, sonicated for 1 h, and centrifuged at 14,000×g for 10 min before being analyzed.

Sample analysis was performed by LC-ESI-MS/MS using an Agilent 6495 triple quadrupole mass spectrometer adapting a method described before [28]. Liquid chromatography was performed with a ZORBAX eclipse plus C18 column (2.1 × 100 mm 1.8 mm, Agilent) heated at 60 °C. Mass spectrometry analysis was performed in positive and negative ion mode with dynamic scheduled multiple reaction monitoring (MRM).

The solvent A consisted of 50% H2O/30% acetonitrile/20% isopropanol (v/v/v) and solvent B consisted of 1% H2O/9% acetonitrile/90% isopropanol (v/v/v), both containing 10 mM ammonium formate. Gradient started with a flow rate of 0.4 ml/min at 10% B and increasing to 45% B over 2.7 min, then to 53% over 0.1 min, to 65% over 6.2 min, to 89% over 0.1 min, to 92% over 1.9 min, and finally to 100% over 0.1 min. The solvent was then held at 100% B for 2.3 min. For column equilibration, solvent B was decreased from 100 to 10% over 0.1 min and held for an additional 0.9 min; flow rate was then switched to 0.6 ml/min for 1 min before returning to 0.4 ml/min over 0.1 min; and finally, solvent B was held at 10% B for a further 0.9 min at 0.4 ml/min.

The following mass spectrometer conditions were used: gas temperature 150 °C, gas flow rate 17 L/min, nebulizer 20 psi, sheath gas temperature 200 °C, capillary voltage 3500 V, and sheath gas flow 10 L/min. Isolation widths for Q1 and Q3 were set to unit resolution (0.7 amu).

18:2 CE concentration was estimated using a standard curve (Supplementary Table 1) and corrected using Quality Controls (QC) consisting of a mix of all samples, injected between samples. The samples were thawed once and no freeze-thaw stability test was performed.

### Statistical Analyses

All the statistical tests and graphs were performed using GraphPad Prism 9 (GraphPad Software). *T*-test was employed for two group mean comparison. Correlation analyses were assessed by Spearman’s rank. ROC curve was estimated for potential 18:2 CE use of NEDA-3 predictor. Multivariate logistic regression model was performed for NEDA-3 ROC curve of 18:2 CE and clinical variables. The threshold for significance was set at 0.05.

## Results

A total of 115 MS patients (2010 McDonald’s revised criteria) and 57 controls were included in the study (Figure S1). At baseline, the mean interval between clinical onset and diagnosis was 51.68 months (SD 75.28). Regarding MRI lesion burden, 64% of patients presented with fewer than 20 T2-hyperintense lesions and 36% with more than 20 T2-hyperintense lesions on the baseline scan. Gadolinium-enhanced MRI was performed in 92 patients (80%), of whom 37 (40%) showed at least one gadolinium-enhancing lesion. All patients were treatment-naïve at the time of inclusion in the study. The clinical characteristics of the participants are described in Table 1.

**Table 1:** Description of the participants. RRMS, relapsing-remitting multiple sclerosis; PPMS, primary progressive multiple sclerosis; EDSS, expanded disability severity score; OCB, oligoclonal bands. Data are shown as mean ± SD unless otherwise stated

	All MS, *N* = 115	RRMS, *N* = 94	PPMS, *N* = 21	Controls, *N* = 57
Age	39.40 ± 10.23	37.81 ± 10.42	44.72 ± 8.58	40.52 ± 9.31
Sex (% Female)	77 (66.96)	70 (74.47)	7 (33.33)	41 (71.93)
EDSS (diagnostic)	1.84 ± 1.34	1.56 ± 1.26	3.19 ± 0.79	
OCB (+/Total)	63/99	50/81	13/18	
Disease duration at diagnosis	51.68 ± 75.28 months	46.14 ± 61.07 months	53.17 ± 112. 25 months	
Gadolinium-enhanced RMI	37	32	5	
T2-hyperintense lesions < 20	74	58	16	
T2-hyperintense lesions > 20	41	36	5	

Regarding DMT exposure over the 10-year follow-up, 75 patients were initially treated with moderate-efficacy agents (42 with interferon, 18 with glatiramer acetate, 5 with dimethyl fumarate, and 2 with teriflunomide), whereas 16 patients received high-efficacy therapy as first-line treatment (5 natalizumab, 5 fingolimod, and 6 anti-CD20). Escalation to high-efficacy DMT due to insufficient response occurred in 26 patients as second-line therapy (12 natalizumab, 4 fingolimod, 9 anti-CD20, and 1 alemtuzumab) and in a further 7 patients as third-line treatment (2 natalizumab, 1 fingolimod, 2 cladribine, 1 anti-CD20, and 1 alemtuzumab).

### 18:2 CE CSF Levels Are Elevated in OCB + Participants

**Fig. 1:**
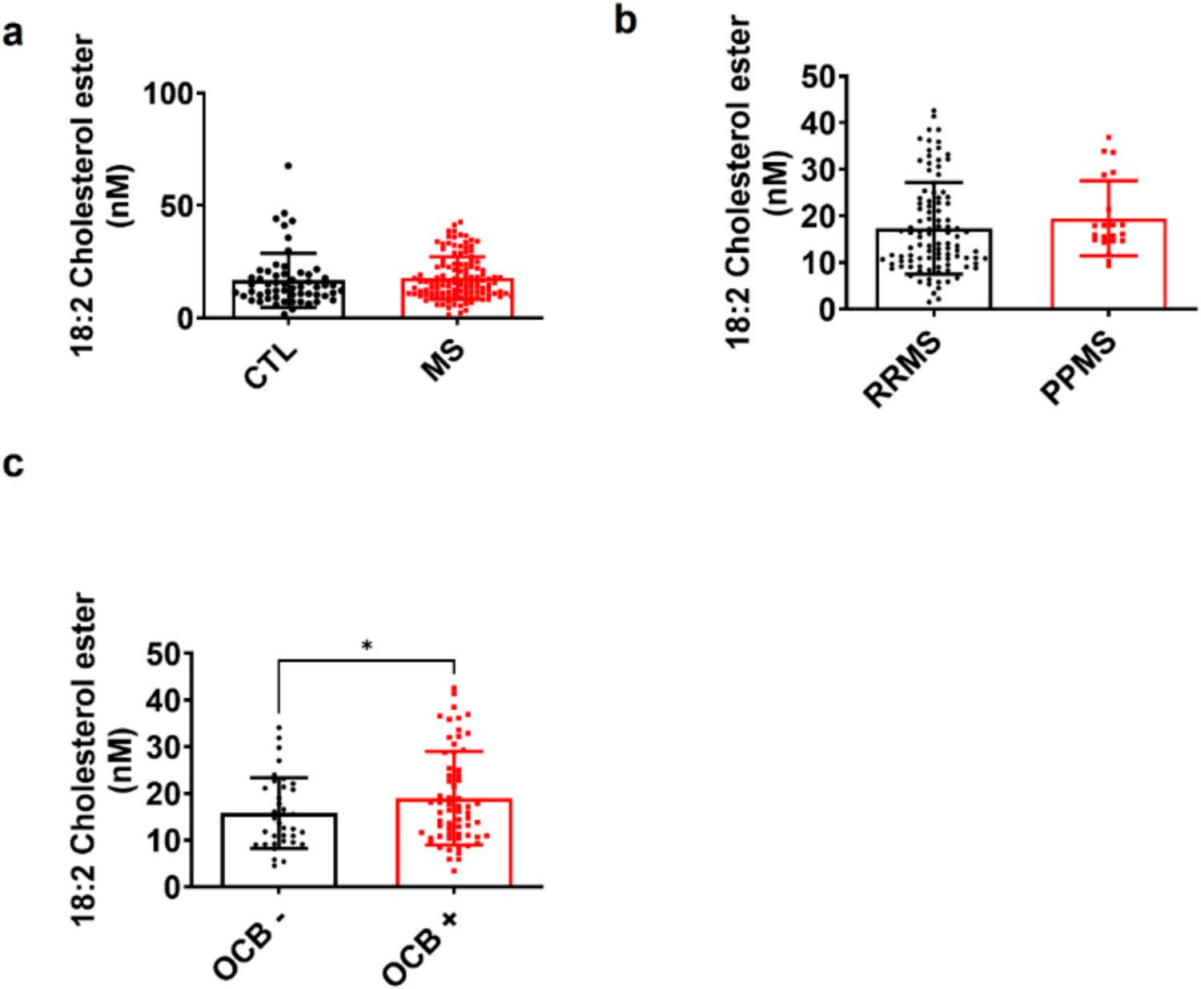
CSF 18:2 CE levels are not useful for MS diagnosis, 95% CI (CTL): 13.52–19.91. 95% CI (MS): 16.01–19.41 nM (**a**), nor for distinguishing between RRMS and PPMS, 95% CI (RRMS): 15.42–19.27 nM. 95% CI (PPMS): 15.82–23.16 nM (**b**). However, within the MS cohort, patients positive for OCBs exhibit higher CSF levels of 18:2 CE 95% CI (OCB-): 13.21–18.33 nM. 95% CI (OCB +): 16.48–21.49 nM. *p* < 0.05, unpaired *t*-test. CE, cholesterol ester; OCB, oligoclonal bands

We wanted to explore the potential use of 18:2 CE as a diagnostic biomarker. We did not find statistically significant differences between the controls and the MS patients, including RRMS and PPMS (Fig. 1a). 18:2 CE was not different between RRMS and PPMS (Fig. 1b). However, MS patients with OCB + had higher levels of the 18:2 cholesterol ester in their cerebrospinal fluid than those with OCB- (Fig. 1c).

### 18:2 CE Is a Promising Long-Term Prognostic Biomarker

**Fig. 2:**
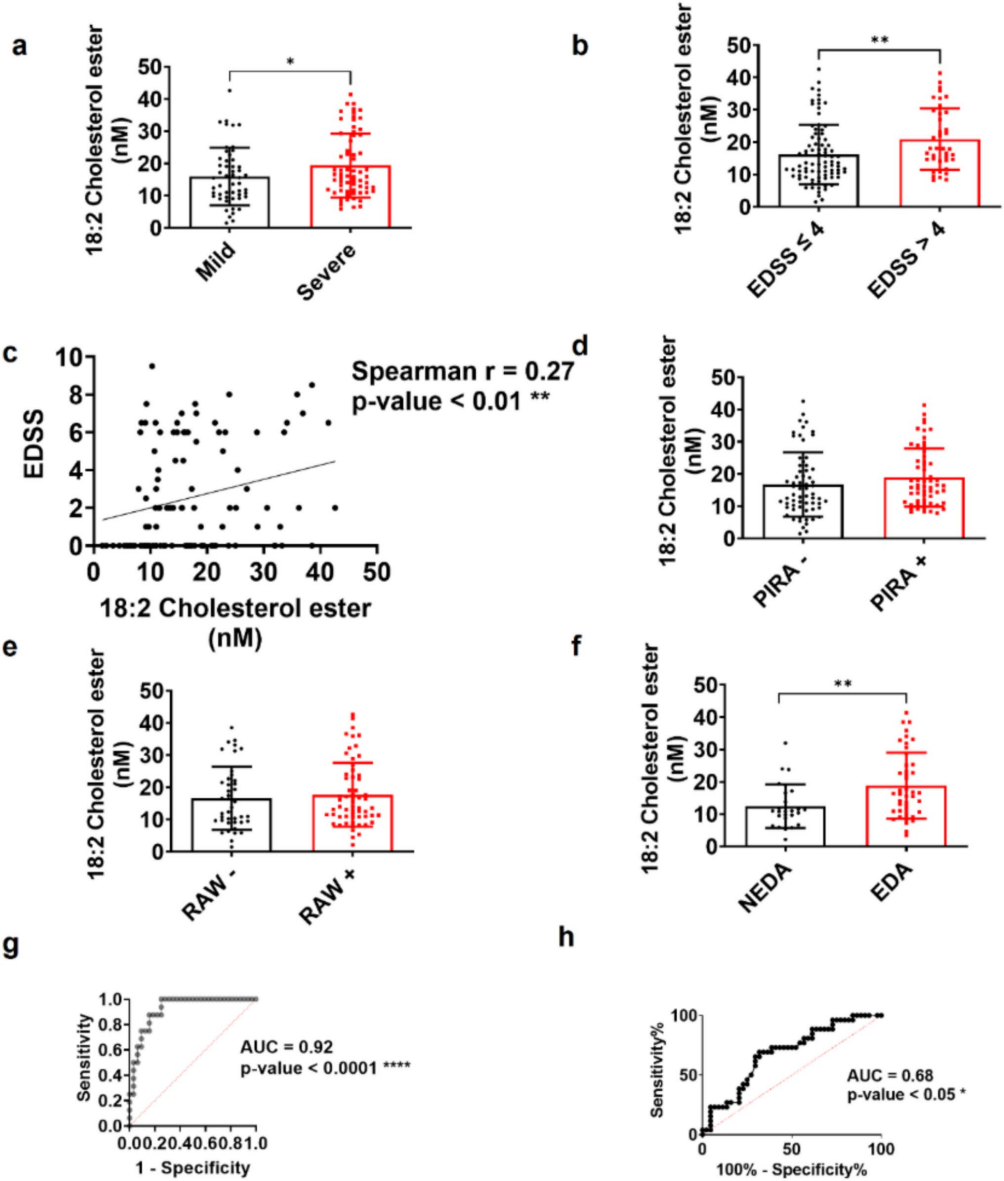
Higher CSF 18:2 CE levels predict a more severe disease course, 95% CI (mild): 13.5–18.49 nM. 95% CI (severe): 16.95–21.81 nM (**a**)**,** and are elevated in patients with EDSS > 4. 95% CI (EDSS < 4): 14.2–18.2 nM. 95% CI (EDSS > 4): 17.9–24.06 nM (**b**). The 10-year follow-up EDSS correlates with diagnostic CSF 18:2 CE levels. 95% CI (slope): 0.01947–0.1313. 95% CI (Y-intercept): 0.1725–2.331. 95% CI (X-intercept): − 113.8– − 1.381 (**c**). However, patients with PIRA, 95% CI (PIRA-): 14.28–19.27 nM, 95% CI (PIRA +): 16.54–21.26 nM (**d**), or RAW, 95% CI (RAW-): 13.53–19.7 nM, 95% CI (RAW +): 15.16–20.28 nM (**e**), do not exhibit increased 18:2 CE levels. Patients with active disease (EDA) 10 years after diagnosis have higher CSF 18:2 CE levels than NEDA-3 patients, 95% CI (EDA): 15.75–21.95 nM. 95% CI (NEDA-3): 9.763–15.22 nM (**f**). Multiple logistic regression incorporating 18:2 CE levels together with diagnostic RAW, location of the diagnostic relapse, EDSS at diagnosis and at 6 months, and OCB presence yielded an ROC AUC of 0.92 (**g**). ROC curve for 18:2 CE in predicting NEDA-3 status (**h**). *p* < 0.05, *p < 0.01, ***p < 0.0001, unpaired *t*-test

When we compared 18:2 CE levels in the total group of MS patients between mild (EDSS ≤ 3 with 10 years of evolution without any progression of disability except for random outbreaks and without high-efficacy treatments) and severe (EDSS ≥ 6 after 10 years of evolution or with high-efficacy treatments over 10 years) forms, we found significant differences, with higher levels in the latter group (Fig. 2a).

Next, we attempted to determine whether 18:2 CE levels were different according to EDSS-only criteria for disease severity (≤ 4 vs > 4). We found that patients with an EDSS score of ≤ 4 had lower levels of 18:2 cholesterol ester than those with EDSS scores of > 4 (Fig. 2b). EDSS score after 10 years of follow-up was significantly associated with 18:2 CE levels in CSF at diagnostic point (Fig. 2c).

We also classified the patients by PIRA in order to check 18:2 CE potential use for its prediction since it could tell us that this lipid might be associated also with relapse independent progression. We did not find differences between PIRA+ and PIRA– patients (Fig. 2d). RRMS patients were also grouped by the presence of RAW, but no significant differences were observed (Fig. 2e).

We also analyzed 18:2 CE levels classifying patients by NEDA-3 criteria. In the same line with other prognostic variables, NEDA-3 patients have lower 18:2 CE levels (Fig. 2f). Since these differences were the highest compared with the other prognostic criteria, we performed a ROC analyses. In a univariate analysis, 18:2 CE alone had an AUC of 0.69, *p* < 0.05. We envisage better prognostic power when combined with other variables, all of them around diagnostic time (Supplementary Table 2). Therefore, we generated a multiple logistic regression model with 18:2 CE, RAW, lesion location, EDSS at diagnostic and 3–6 months after it and the presence of OCB. This combination reported an AUC of 0.92 p < 0.0001 (Fig. 2g).

### 18:2 CE Is Elevated in Patients with Spinal Lesions

**Fig. 3:**
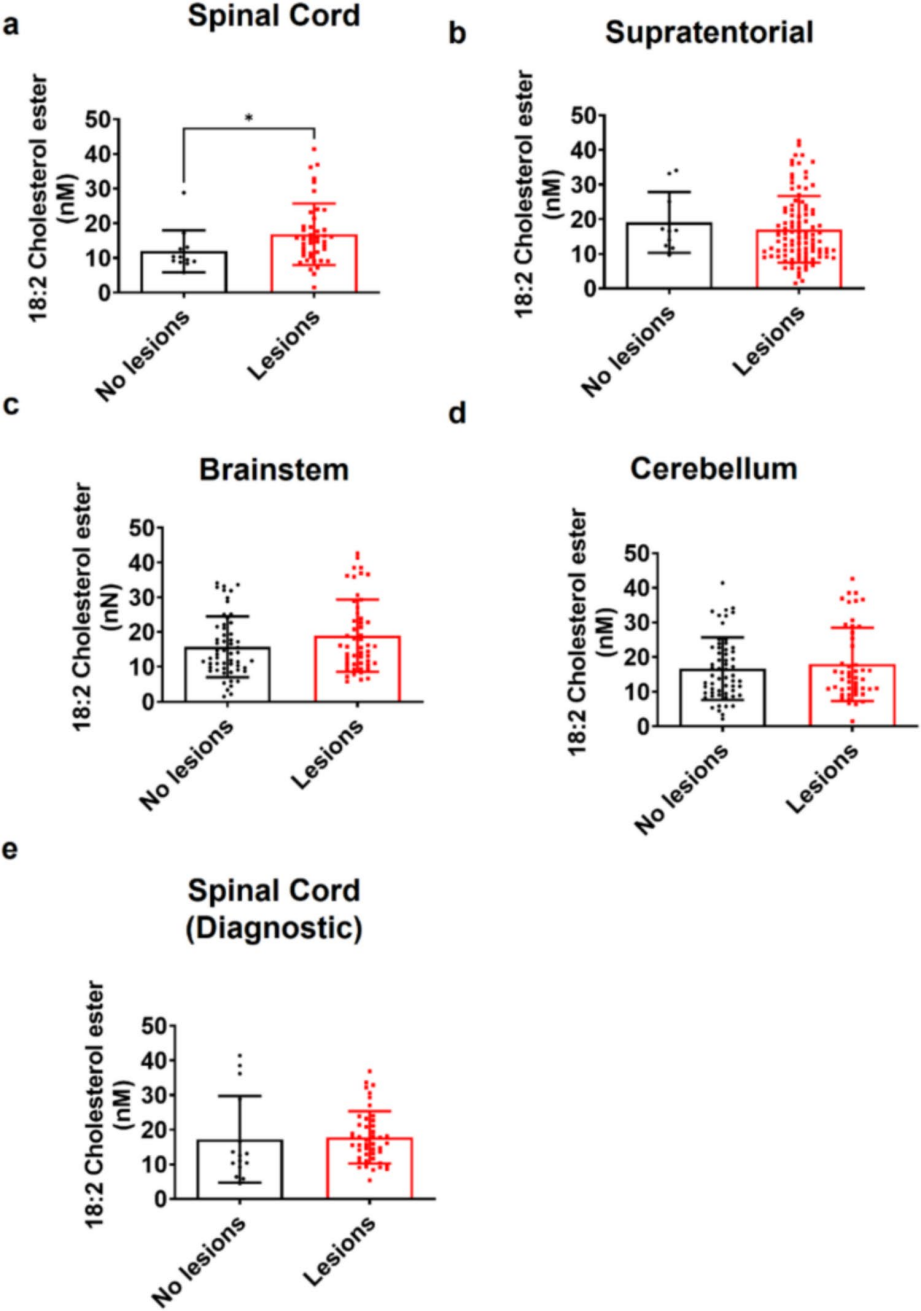
Elevated CSF 18:2 CE levels are associated with the presence of prospective spinal cord lesions, 95% CI (no lesions): 8.089–15.76 nM, 95% CI (lesions): 14.1–19.56 nM (**a**). However, no association is observed with lesions in the supratentorial region, 95% CI (no lesions): 12.82–25.34 nM, 95% CI (lesions): 15.2–19 nM (**b**); brainstem, 95% CI (no lesions): 14.3–18.99 nM, 95% CI (lesions): 14.86–21 nM (**c**); or cerebellum, 95% CI (no lesions): 13.4–18.07 nM, 95% CI (lesions): 16.04–21.84 nM (**d**). Interestingly, spinal cord lesions at diagnosis are not associated with increased CSF 18:2 CE levels, 95% CI (no lesions): 10.29–24.15 nM, 95% CI (lesions): 15.5–19.99 nM (**e**). *p* < 0.05, unpaired *t*-test

When we studied 18:2 CE levels with respect to the radiological locations of demyelinating lesions, we found that MS patients who did not experience spinal cord injuries had lower levels of 18:2 CE than those with spinal cord lesions throughout the 10-year development of the disease (Fig. 3a). We did not find any statistically significant differences regarding the other territories studied: supratentorial, brainstem, and cerebellum (Fig. 3b–d, respectively). We similarly found no differences regarding whether the relapse that prompted the study was spinal or had another location (Fig. 3e).

### DMT Efficiency Is Associated with 18:2 CE

**Fig. 4:**
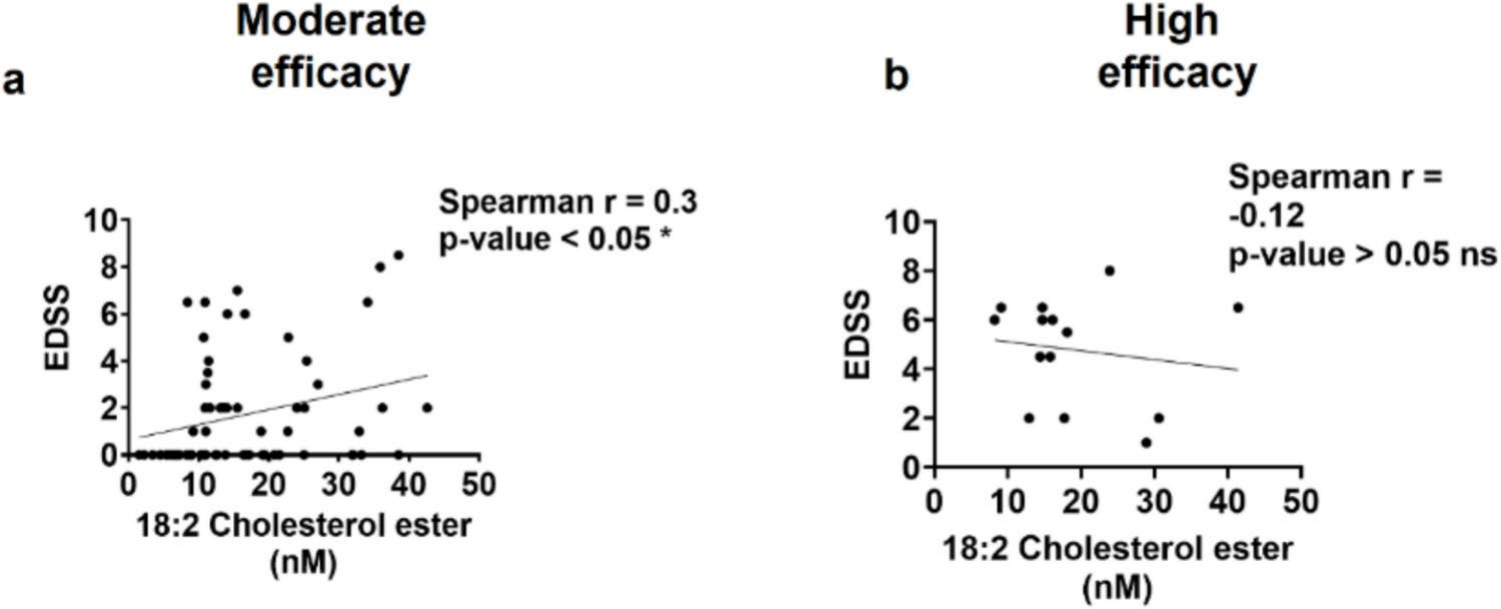
CSF 18:2 CE levels predict EDSS changes during follow-up in patients treated with moderate-efficacy DMTs, 95% CI (slope): 0.007611–0.1208, 95% CI (Y-intercept): − 0.4475–1.746, 95% CI (X-intercept): − 210.9–4.032 (**a**), but not in those receiving high-efficacy therapies, 95% CI (slope): − 0.1845–0.1117, 95% CI (Y-intercept): 2.371–8.586, 95% CI (X-intercept): 43.75– + infinity (**b**). *p* < 0.05, unpaired *t*-test. n.s., not significant

We analyzed potential interactions with DMT and the choice of high- or moderate-efficacy and changing from moderate to high-efficacy or vice versa as a second or third option. We evaluated the correlation of EDSS after follow-up in an 18:2 CE. We obtained a positive correlation when a moderate efficacy DMT was administered as the first treatment (Fig. 4a). When the first DMT was high-efficacy, follow-up EDSS and 18:2 CE did not correlate (Fig. 4b).

## Discussion

In this study, we aim to analyze the potential use of 18:2 CE in the CSF of MS patients at diagnostic time as a biomarker of diagnostic and prognostic. We used long-term follow-up (10 years) CSF samples from diagnostic. One of the most studied biomarkers in MS and other neurological diseases is the neurofilament light chain (Nfl) levels in CSF, and serum Nfl is a reflection of acute axonal damage [29, 30]. However, Nfl is a non-cell specific marker, elevated in neurodegenerative diseases, not specific of MS. Moreover, Nfl is not a therapeutic target and does not inform us about disease mechanisms. Note than Nfl prognostic value for NEDA-3 status decline in the following years from baseline measure [31], being not useful for predictions over 4 years after diagnostic, potentially influenced using high-efficacy DMTs [32]. Another recently highlighted biomarker of interest is glial fibrillary acidic protein (GFAP). GFAP is suggested to serve as a biomarker indicating damage to astrocytes and reactive astrogliosis in various inflammatory and neurodegenerative diseases [33, 34].

Serum GFAP (sGFAP) levels were associated with higher risk of 6-month confirmed disability progression in MS [35]. In recent studies, sGFAP was associated with 10-year EDSS. Based on these observations, the authors wanted to study the effect of adding the early determination of GFAP to the predictive models of progression, yielding only modest results compared to previous clinical and radiological predictive models [36].

While MRI constitutes an essential tool for diagnosis and monitoring of multiple sclerosis, its long-term prognostic accuracy remains limited. There is scarce evidence supporting the ability of MRI-derived parameters to predict disease evolution beyond 10 years. Moreover, expert visual inspection of MRI images is laborious and time-consuming, bringing about the need for automated tools to extract meaningful biomarkers.

In this context, the identification of a biochemical biomarker such as 18:2 CE offers several advantages. Being operator-independent and objectively quantifiable, 18:2 CE may represent a more reliable and reproducible measure of disease activity. Its robust association with future NEDA-3 loss supports the notion that metabolic alterations reflected by 18:2 CE could capture pathological processes not readily detectable by MRI, thereby complementing imaging- and clinically derived prognostic models.

Our group has demonstrated the potential use of CSF lipidomics for MS biomarkers [25]. Our previous data (not published) pointed to 18:2 CE in CSF as a biomarker of poor prognostic. Lipidic species are especially relevant for MS biomarkers of injury and may be a lack or deficiency of remyelination because demyelination and a lack of repair are relevant for disease progression [37] and a potential therapeutic target. Of note, brain contains 20% of whole body’s cholesterol, being the most enriched organ [38]. Myelin is specially enriched with lipids and cholesterol plays a key role in membrane stability. Cholesterol turnover in CSF is very slow, with a half-life of 0.5—5 years. Nevertheless, cholesterol levels can be altered in disease and aging [39].

Excess cellular cholesterol is implicated in chronic microglial activation and smouldering phenomena [4]. It can be esterified and directed to intracellular lipid droplets [39]. The lipid droplets play a crucial role by not only shielding cells from excessive lipid levels but also serving as repositories for cholesteryl esters and triglycerides. Through the process of lipolysis, cholesterol esterase can convert back the stored molecules into free cholesterol and fatty acids as needed by the cell for functions like cell communication, constructing cell membranes, and energy generation [40]. On the other hand, free cholesterol in plasma and CSF can be transported to lipoprotein (apoE) via esterification mediated by LCAT [7, 8]. Alterations in these processes could hamper membrane synthesis, especially relevant for remyelination.

Our data does not support the diagnostic use of 18:2 CE because there are not statistically significant differences comparing MS and control 18:2 CE levels in CSF at diagnostic point. However, we postulate 18:2 CE as a potential long-term prognostic biomarker. 18:2 CE accumulation in severe MS may be associated with factors such as altered cholesterol esterase activity [41, 42] or increased myelin destruction [43]. However, in our study 18:2 CE levels are not influenced by the presence of RAW in the episode that prompted the CSF study and not differ when comparing controls and all MS cohort. Moreover, it was also not associated with the episode being spinal, or with the presence of asymptomatic lesions in the first MRI.

These findings may reflect increased LCAT activity. Previous studies demonstrated an increase of plasmatic LCAT in MS patients, especially in rapidly progressive cohort [44]. Notably, in this case, LCAT was measured in plasma, not in CSF, so its relationship with the relapse was not possible to assess. Circulating cholesterol ester levels associated with LCAT activity are also altered in Alzheimer’s disease in both plasma and CSF, postulating LCAT activity as a potential target for neurodegeneration [45]. Defect on cholesterol ester uptake could also explain a high amount in CSF. An opposite finding is described in AD: a decrease in CSF [45] together with an excess in most vulnerable brain tissue [46]. One of the processes that could be impaired by defective cholesterol ester circulation in CSF is neuronal synaptogenesis, which depends on glia derived cholesterol forming lipoproteins [47]. Disturbances in cholesterol trafficking are associated with neurodegeneration [7]. Interestingly, an excess of cholesterol derived from myelin debris is associated with a lack of remyelination [48]. Restoring the capacity of cells to remove circulating cholesterol could be useful for regenerative medicine in the CNS [48]. 18:2 CE could also be useful as a candidate biomarker for cholesterol metabolism-related therapies. However, further evidence is required to establish any causal relationship between 18:2 CE alterations and multiple sclerosis pathology. One possible explanation involves deficient myelin debris clearance or altered sterol metabolism within microglial cells, which could impair cholesterol recycling and contribute to remyelination failure. Alternatively, dysregulation of LCAT activity in the CSF may lead to increased cholesterol esterification. Future mechanistic studies addressing these hypotheses will be essential to elucidate the biological pathways underlying 18:2 CE dysregulation in MS.

In our study, we observed that CE 18:2 levels at diagnosis were associated with long-term NEDA-3 status; however, we did not find a significant association between CE 18:2 and PIRA in our cohort. This apparent discrepancy may be partly explained by methodological constraints: PIRA was assessed retrospectively, and progression was defined solely by EDSS worsening, without incorporating more sensitive functional or cognitive measures such as timed walking tests, the Symbol Digit Modalities Test, or the 9-Hole Peg Test, which were not routinely performed in our clinical follow-up 10 years ago and were therefore unavailable for this analysis. In addition, although the ROC performance for predicting NEDA-3 is encouraging, NEDA-3 remains a composite endpoint with recognized limitations, particularly its dependence on MRI monitoring frequency and relapse ascertainment, which can introduce variability and potentially underestimate subclinical activity. Taken together, these findings support a potential prognostic role for CE 18:2 in long-term disease control, while highlighting the need for prospective validation using multidimensional progression outcomes.

Remarkably, 18:2 CE levels were found to be elevated in MS patients with spinal cord lesions throughout the disease, indicating a potential association between lipid metabolism and the specific anatomical involvement of demyelinating lesions. This insight highlights the need for further exploration into the regional variations of lipid profiles in MS, particularly concerning spinal cord lesions. Patients initially treated with highly effective disease-modifying treatments did not show a positive correlation between 18:2 CE levels and EDSS. Therefore, we interpret that using high efficacy drugs as a first option may be associated with a modified prognosis of the disease. On the other hand, patients treated with high efficacy drugs as a second or third option do exhibit a positive correlation between 18:2 CE levels and EDSS. Consequently, using high efficacy drugs as a second or third option may not be associated with a modified prognosis of the disease. This suggests that 18:2 CE levels could potentially help identify patients who may require high-efficacy treatments from the beginning due to their risk of long-term progression. These findings are exploratory and hypothesis-generating and should be interpreted cautiously pending prospective, multicenter validation.

However, our study has several limitations. Firstly, a retrospective assessment has been conducted based on the increase in scores on the EDSS scale, and prospective measures could not be taken for the 25-step test (T25-FW) or the Nine-Hole Peg Test (9-HPT) and the Symbol Digit Modalities Test (SDMT). Additionally, we are aware that the approach to treating patients with MS has changed compared to 10 years ago, resulting in a small sample of patients treated with high-efficacy drugs as a first option. Moreover, brain volume metrics were not available, as at the time the MRIs were acquired, our centre did not have validated/standardized volumetric quantification techniques. Lastly, the methodology used in our study allows us to quantify the number of 18:2 CE, but it was not possible to differentiate between the proportion of CE found in a free form and that present in lipid droplets. Consequently, we cannot assign the observed associations to a specific CE pool, and lipid-droplet–bound CE may contribute to the prognostic signal. Expanding studies to attribute pool-specific signals could help to improve our understanding of this pathway. Finally, we measured 18:2 CE only, although the combined assessment of sNfL and GFAP might improve sensitivity and clinical utility. Prospective head-to-head and combinatorial analyses against sNfL/GFAP would be of interest, given that sNfL/GFAP alone are weak long-term prognosticators [49].

The use of NEDA-3 as the outcome for ROC analyses provides a comprehensive assessment of this biomarker’s ability to predict prognosis because it includes clinical relapses, MRI evidence of disease activity, and disability worsening.

Prospective validation of these findings is essential for the eventual translation of CSF 18:2 CE into clinical practice, offering a promising avenue for improved patient management in multiple sclerosis; however, the present results should be regarded as exploratory and hypothesis-generating, pending multicenter prospective validation.

## Supplementary Information

Below is the link to the electronic supplementary material.

ESM 1 (PDF 40.8 KB)

ESM 2 (XLSX 15.4 KB)

ESM 3 (XLSX 7.50 KB)

## Data Availability

Data are available on reasonable request. The data that support the findings of this study are available from the corresponding author on reasonable request.
