# Supplementary figures and images for: 18:2 Cholesterol Ester is a Novel Prognostic Biomarker of Disease Progression in Multiple Sclerosis
Source: Mol Neurobiol. 2026 Jun 17;63(1):698. doi: 10.1007/s12035-026-05915-8 (PMC13275756; doi:10.1007/s12035-026-05915-8)

## Flow Diagram

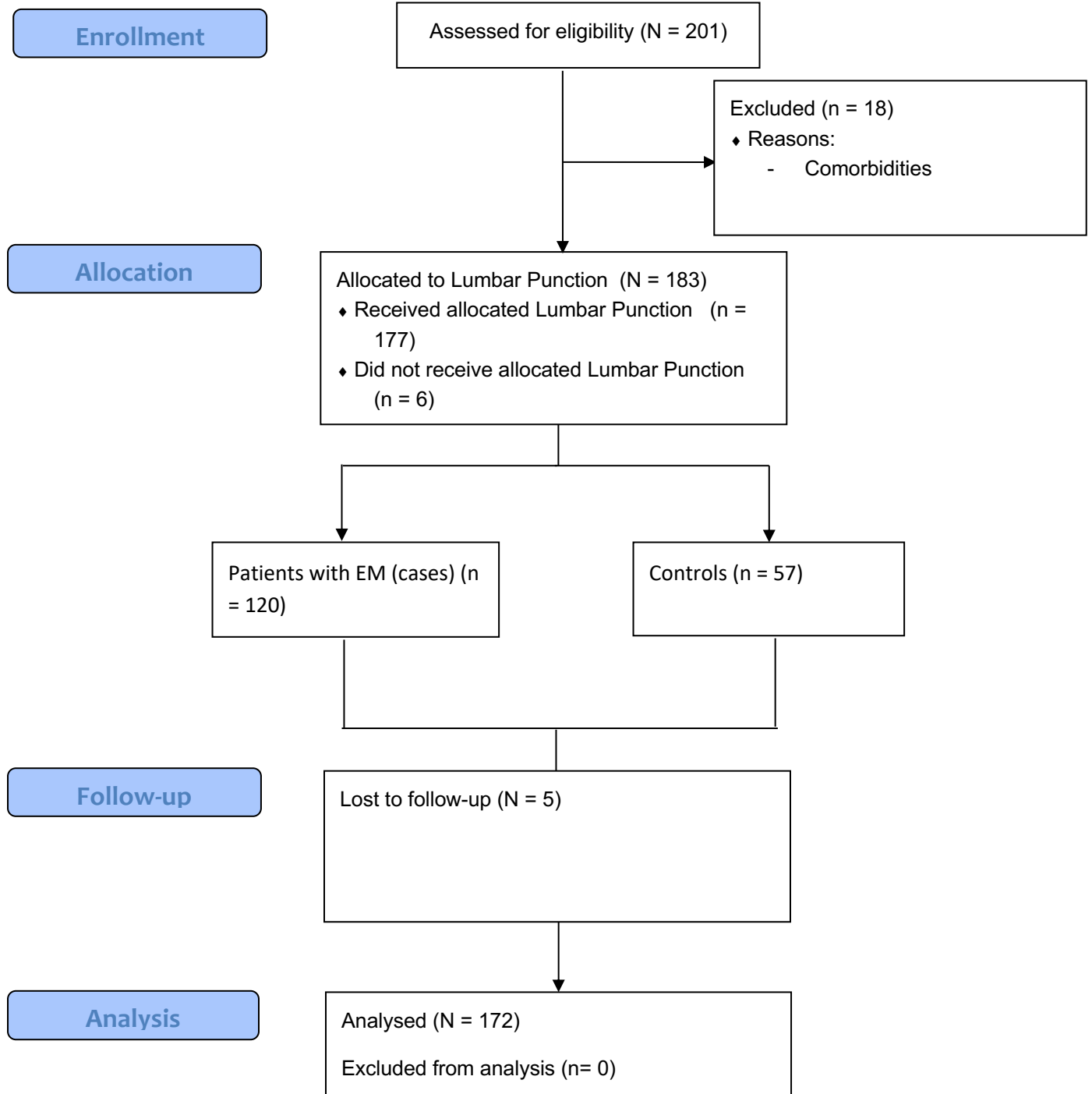

Supplement: Supplementary file 1 — (PDF 40.8 KB) [file 12035_2026_5915_MOESM1_ESM.pdf]
